# Diverging Safety Signals: A Trend Analysis of Suspected Adverse Drug Reactions Reporting for Spinal Muscular Atrophy Therapies in the European Union

**DOI:** 10.3390/neurolint17100165

**Published:** 2025-10-08

**Authors:** Andrej Belančić, Petar Mas, Ivana Stević, Dinko Vitezić, Slobodan Janković

**Affiliations:** 1 Department of Basic and Clinical Pharmacology and Toxicology, Faculty of Medicine, University of Rijeka, 51000 Rijeka, Croatia; dinko.vitezic@medri.uniri.hr; 2 Agency for Medicinal Products and Medical Devices, 10000 Zagreb, Croatia; Petar.Mas@halmed.hr; 3 Faculty of Pharmacy, University of Belgrade, 11000 Belgrade, Serbia; ivana.stevic@pharmacy.bg.ac.rs; 4 Faculty of Medical Sciences, University of Kragujevac, 34000 Kragujevac, Serbia

**Keywords:** spinal muscular atrophy, pharmacovigilance, adverse drug reactions, drug safety, nusinersen, onasemnogene abeparvovec, risdiplam

## Abstract

**Background/Objectives**: The approval of disease-modifying therapies has significantly improved outcomes for patients with spinal muscular atrophy (SMA), yet their long-term safety profiles remain under continuous evaluation. This study aimed to assess trends in the reporting of suspected adverse drug reactions (ADRs) associated with nusinersen, onasemnogene abeparvovec, and risdiplam across the European Union. **Methods**: We conducted a secondary analysis of annual suspected ADR data reported to EudraVigilance from 2017 to 2024 for the three approved disease-modifying therapies for SMA. On top of general reporting trend, specific adverse reactions of interest included post-lumbar puncture syndrome for nusinersen, liver toxicity and elevated serum troponin for onasemnogene abeparvovec, and respiratory and gastrointestinal reactions for risdiplam. Joinpoint regression analysis was used to evaluate annual percent changes and identify statistically significant trend segments for each medicine. **Results**: The reporting of suspected ADRs for nusinersen showed an initial increase, followed by a significant decline after 2019. Onasemnogene abeparvovec exhibited a continued but decelerating increase in suspected ADRs, while risdiplam demonstrated a consistent upward trend across all reported reactions. **Conclusions**: Diverging patterns in adverse reaction reporting suggest a stabilizing safety profile for nusinersen and potential emerging safety signals for risdiplam and onasemnogene abeparvovec, underscoring the need for ongoing continued pharmacovigilance (e.g., post-authorization studies and spontaneous reporting).

## 1. Introduction

Spinal muscular atrophy (SMA) is a rare, autosomal recessive neuromuscular disorder characterized by progressive degeneration of motor neurons, leading to muscular atrophy and severe physical disability [1]. Until recently, the therapeutic approach to SMA was largely supportive, with no options to alter the disease’s trajectory. However, the advent of disease-modifying treatments has revolutionized SMA care, markedly improving motor function and survival in affected individuals [2,3].

Nusinersen, an intrathecally administered antisense oligonucleotide, became the first approved disease-modifying treatment in Europe, following the ENDEAR and CHERISH trials, which demonstrated clinically significant improvements in motor milestones and survival among infants and children with SMA. It received European Medicines Agency (EMA) approval in May 2017 [4,5,6]. Onasemnogene abeparvovec, a one-time intravenous gene replacement therapy targeting SMN1 gene deficiency, followed with conditional approval in May 2020—converted to standard authorization in 2022—supported by the START and STR1VE trials [7,8,9,10]. Most recently, risdiplam, a daily oral SMN2 splicing modifier, gained EMA authorization in March 2021, with evidence from the FIREFISH and SUNFISH trials confirming its efficacy and safety across pediatric and adult SMA populations [11,12,13].

While these therapies represent substantial therapeutic advancements, both long-term safety and efficacy remain ongoing concerns. All three agents are subject to mandated post-authorization efficacy studies (PAES) to further characterize long-term efficacy [14]. Long-term safety is further characterized through long-term extension studies, registry studies and routine pharmacovigilance through spontaneous adverse drug reactions reporting, as described within structured Risk Management Plans (RMPs) mandated by regulatory authorities [15]. EudraVigilance (EV), the European database for suspected adverse drug reaction reports, serves as a critical tool for post-marketing surveillance, enabling the detection of evolving safety signals [16,17].

Monitoring trends in adverse reaction reporting over time can offer insights into the real-world safety of these therapies. A consistent decline in serious adverse reaction reports concurrent with increasing drug utilization may indicate a favorable safety profile. Conversely, rising adverse reaction trends could signal emerging safety concerns warranting further investigation [18]. In this context, we aimed to analyze temporal patterns in adverse drug reaction reporting for nusinersen, onasemnogene abeparvovec, and risdiplam in the European Union to identify discrepancies suggestive of potential safety signals or pharmacovigilance gaps.

## 2. Materials and Methods

The study was designed as secondary research, utilizing data on the number of all suspected adverse reactions and some specific adverse reactions associated with drugs used to treat Spinal Muscular Atrophy (SMA). This data was obtained from EudraVigilance, the European database of suspected adverse drug reactions (ADRs) reports, which has been established and maintained by the European Medicines Agency (EMA) since 2002 [19]. Data was accessed via publicly available adrreports.eu portal, which allows access to spontaneously reported suspected ADRs held in EudraVigilance since the drug was authorized for use in the EEA and does not include cases from clinical or non-interventional studies. This type of research did not require ethics committee approval.

The suspected ADRs were analyzed for three drugs that have been approved for the treatment of SMA in European Union countries: nusinersen, onasemnogene abeparvovec, and risdiplam. The primary outcome variable for the study was the total count of reported ADRs for all three drugs on an annual basis, starting from the initiation of EudraVigilance reporting. The secondary outcome variables included the annual rate of post-lumbar puncture syndromes for nusinersen, the annual rates of liver damage and serum troponin increase associated with onasemnogene abeparvovec, and the annual rates of respiratory and gastrointestinal adverse reactions in patients treated with risdiplam. Cases were identified by using following standardized MedDRA terms: PTs “Post lumbar puncture syndrome”, “Troponin I increased”, “Troponin increased” and “Troponin T increased”, SOCs “Respiratory, thoracic and mediastinal disorders” and “Gastrointestinal disorders”, and SMQ “Liver related investigations, signs and symptoms”. Specific suspected ADRs were selected a priori on the basis of clinical relevance and previously reported signals in Belančić et al. manuscript entitled Post-Marketing Safety of Spinal Muscular Atrophy Therapies: Analysis of Spontaneous Adverse Drug Reactions from EudraVigilance [20]. The reporting year served as the independent variable.

Trends in the annual reporting rates for these three drugs were analyzed using Joinpoint Trend Analysis Software, version 5.2.0, which is issued and maintained by the National Cancer Institute of the USA. This regression software divides the trendline into an optimal number of segments connected by “joinpoints,” testing the significance of the trend within each segment. The significance of the trends was evaluated using a Monte Carlo Permutation method, and the annual percent change in the rates was computed [21,22]. The significance level was set at a *p*-value of ≤0.05 for the null hypothesis. The null hypothesis was that there were no significant temporal trends in the observed data.

## 3. Results

**Figure 1:**
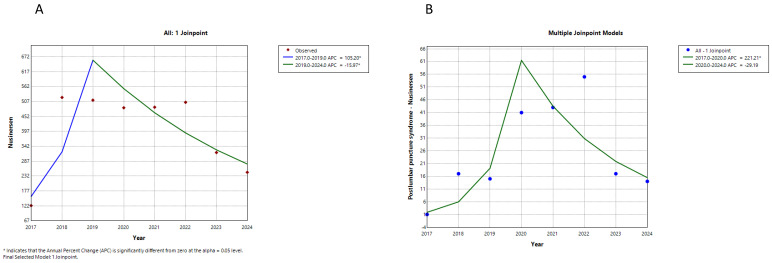
Trends of reporting suspected adverse drug reactions (ADRs) associated with nusinersen. Note: (**A**) overall, (**B**) postlumbar puncture syndrome.

**Figure 2:**
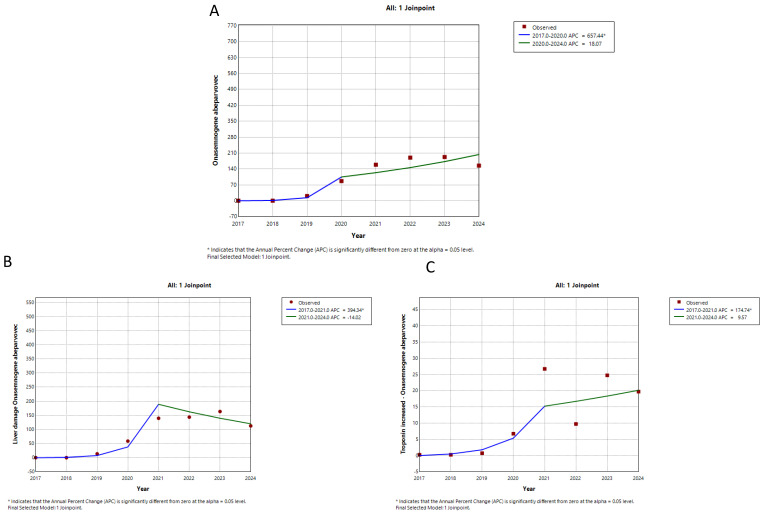
Trends of reporting suspected adverse drug reactions (ADRs) associated with onasemnogene abeparvovec (OA). Note: (**A**) overall, (**B**) liver damage, (**C**) troponin increase.

**Figure 3:**
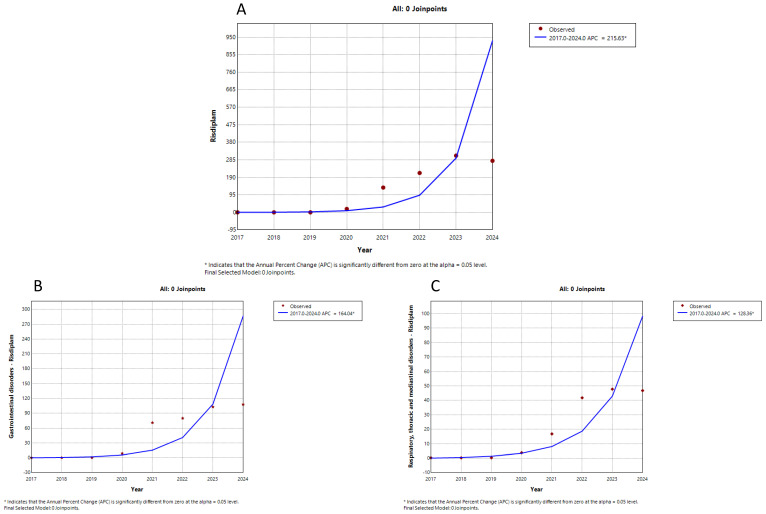
Trends of reporting suspected adverse drug reactions (ADRs) associated with risdiplam. Note: (**A**) overall, (**B**) gastrointestinal disorders, (**C**) respiratory, thoracic and mediastinal disorders.

Trends in the counts of suspected adverse drug reactions (ADRs) per year were analyzed for three drugs approved for the treatment of spinal muscular atrophy (SMA): nusinersen, onasemnogene abeparvovec, and risdiplam. This analysis covered the years 2017 to 2024, depending on the availability of data from Eudravigilance, the European database. The data were extracted from the database up to 31 December 2024. The trends for nusinersen suspected ADRs are illustrated in Figure 1, while the trends for onasemnogene abeparvovec and risdiplam suspected ADRs are presented in Figure 2 and Figure 3, respectively. The annual percent change in the counts and the significance of the trends for each drug are summarized in Table 1.

The trends for suspected ADRs related to nusinersen and onasemnogene abeparvovec each exhibited two segments. The first segment showed a significant increase for both drugs, while the second segment revealed a significant decrease for nusinersen and an insignificant increase for onasemnogene abeparvovec. Trends for specific suspected ADRs for these two drugs closely followed the overall trends for all suspected ADRs. In contrast, the total suspected ADRs for risdiplam, along with gastrointestinal and respiratory suspected ADRs, displayed only one upward trend segment (see Figure 1, Figure 2 and Figure 3, and Table 1). Therefore, while the reporting of suspected ADRs for nusinersen began to decline after peaking in 2019–2020, the reporting of onasemnogene abeparvovec continues to grow, albeit at a slower rate starting in 2020, while reporting for risdiplam is steadily increasing.

## 4. Discussion

The trend analysis of the total number of reports of suspected ADRs for all three observed drugs showed a statistically significant initial growth trend, which was highest for OA (APC = 657.44), followed by risdiplam (APC = 215.63), and lowest for nusinersen (APC = 105.20). On the other hand, trend reporting peak for specific suspected ADRs for OA and nusinersen is observed to be delayed by one year in relation to the peak of total suspected ADRs, while with risdiplam, there is only a growing trend in both total and specific suspected ADRs reporting.

It is interesting that the drug nusinersen shows the “Weber effect”, i.e., the peak trend of reports of total suspected ADRs at the end of the second year after obtaining the license. The drugs OA and risdiplam do not show the “Weber effect”. Our results align with those of other publications, which have shown that the Weber effect is not observed with all drugs [23,24,25]. For nusinersen, after an upward trend, a statistically significant decrease in adverse reaction reporting was observed (APC = −15.97). The reasons for this phenomenon may be that the drug has been on the market longer, the seriousness of suspected ADRs, less utilization of the drug (eg. switch to another drug), or no new suspected ADRs are seen, which was seen in other studies [26].

Our results showed a constant increase in the trend of reports with risdiplam, unlike OA or nusinersen, where the trend may be influenced by the drug switch [20] or the method of drug administration, i.e., the trend of reports of gastrointestinal suspected ADRs is higher with oral forms. For instance, a study with semaglutide showed that gastrointestinal suspected ADRs with oral forms is more common [27].

Specific suspected ADRs were also analyzed for all three drugs, selected based on the work of Belančić et al. [20]. It should be pointed out that suspected ADRs for risdiplam from the group Respiratory, thoracic and mediastinal disorder, which had a growth trend during the entire observed period, was not listed in the summary of the characteristics of the drug [28]. Also, in the work of Belančić et al., it is emphasized that it is necessary to make a distinction between whether the event is an unwanted effect of the drug or a consequence of the disease [20].

Newer scientific publications focusing on the safety aspects of drugs used in SMA treatment have identified new ADRs or unexpected adverse reactions that were not reported during clinical trials, such as thrombotic microangiopathy or fatal cases of acute liver failure in OA, or cutaneous vasculitis in risdiplam [28,29,30,31]. Also, those newly identified reactions should be taken into account when we talk about the reporting trends, because depending media attention [32] and communication towards healthcare professionals via different channels (e.g., Direct Healthcare Professional Communication, DHPC) regarding safety concerns may increase reporting rate by raising awareness about new ADRs [33].

To the best of our knowledge, this is among the first studies to investigate trends in the reporting of suspected ADRs associated with therapies approved for the treatment of SMA, examining reporting trends both for total and specific suspected ADRs. However, several limitations inherent to secondary data analysis must be acknowledged. The data were derived from the EV database, which includes reports limited to countries within the European Economic Area, along with serious cases from non-EEA countries. As with all spontaneous reporting systems, underreporting and reporting bias are potential concerns, and information on drug utilization or the number of treated patients is unavailable. Furthermore, database contains all suspected ADRs regardless of their causality assessment and no formal causality assessments were conducted as part of this study therefore some reported ADRs may not be directly attributable to the suspected drugs [20]. Potential confounding factors—such as disease progression, polypharmacy, and differential monitoring practices—may also have influenced the observed ADR patterns. Additional methodological limitations apply. The analysis period (2017–2024) was determined by data availability in EV, yet the year of marketing authorization approval differed across the three drugs [nusinersen (2017), OA (2020), and risdiplam (2021)], potentially contributing to the apparent absence of a Weber effect for the latter two agents. Other pharmacovigilance databases, such as the U.S. Food and Drug Administration Adverse Event Reporting System (FAERS) and the Canada Vigilance Adverse Reaction Online Database, were not included within the scope of this study.

## 5. Conclusions

The analysis of reporting trends for nusinersen displayed an expected pattern of a statistically significant decline in ADR reporting approximately two years post-marketing, consistent with the “Weber effect”. This observed trend may be attributable to the approval of other medicinal products for SMA and less utilization of nusinersen.

In contrast, the reporting trends for OA and risdiplam did not exhibit a similar decline. Notably, risdiplam showed a sustained increase in ADR reporting rates, which can be explained by continuous switch from other medicinal products to risdiplam—an oral formulation that is potentially associated with a higher occurrence of gastrointestinal ADRs compared with pharmaceutical forms/routes of administration.

The observed constant increase in ADR reporting rates from MedDRA SOC Respiratory, thoracic and mediastinal disorders for risdiplam warrants further investigation to determine whether this reflects the underlying disease progression or signals an emerging safety concern. These findings underscore the critical importance of robust post-authorization pharmacovigilance, including long-term safety monitoring through spontaneous ADR reporting and dedicated post-marketing studies. 

## Figures and Tables

**Table 1:** Trend segments, annual percent change, and significance of the trend segments for suspected adverse drug reactions (ADRs).

Medicine	Trend Segment	Annual Percent Change (%)	*p*-Value
***Nusinersen all suspected ADRs***	1	105.2	**0.004**
	2	−16.0	**0.016**
Nusinersen—postlumbar puncture syndrome	1	221.2	**0.000**
	2	−29.2	0.083
***Onasemnogene abeparvovec all suspected ADRs***	1	657.4	**0.000**
	2	18.1	0.567
Onasemnogene abeparvovec—suspected ADRs affecting liver	1	394.3	**0.000**
	2	−14.0	0.669
Onasemnogene abeparvovec—troponin increase	1	174.7	**0.033**
	2	9.6	0.775
***Risdiplam all suspected ADRs***	1	215.6	**0.000**
Risdiplam gastrointestinal suspected ADRs	1	164.0	**0.000**
Risdiplam respiratory suspected ADRs	1	128.3	**0.000**

## Data Availability

Available upon reasonable request sent to the corresponding author.
